# Endocapillary proliferative glomerulonephritis associated with Q fever endocarditis: A case report

**DOI:** 10.1002/ccr3.9473

**Published:** 2024-10-21

**Authors:** Craig Peter Coorey, William James, Venkat Manickavasagam, Jason Chen, Sanjeev Baweja

**Affiliations:** ^1^ Department of Renal Medicine Lismore Base Hospital Lismore New South Wales Australia; ^2^ School of Medicine University of Western Sydney Campbelltown New South Wales Australia; ^3^ Faculty of Medicine and Health The University of Sydney Camperdown New South Wales Australia; ^4^ Department of Anatomical Pathology Royal North Shore Hospital St Leonards New South Wales Australia

**Keywords:** Infectious disease, nephrology

## Abstract

Acute Coxiella burnetti (Q fever) infection is known to activate the autoimmune inflammatory response. We report a rare case of glomerulonephritis associated with the Coxiella infection. An elderly male first presented with recurrent fevers of unknown origin and was subsequently diagnosed with Q fever infection and treated with doxycycline. He represented 2 months later with an acute kidney injury and active urinary sediment. Renal biopsy demonstrated a mesangiopathic pattern with staining for both C1q and IgM, raising possibilities of infection‐related glomerulonephritis, C1q nephropathy and lupus nephritis. This case demonstrates glomerulonephritis as a complication of Q fever infection and the differential diagnosis and workup up that needs to be considered in cases of chronic Q fever.

## Graphical abstract

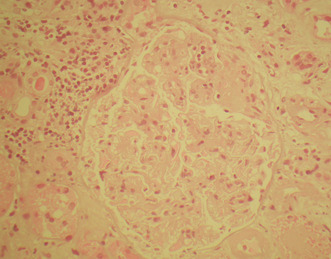

## 1 INTRODUCTION

Q fever is a zoonotic infection caused by a pleomorphic coccobacillus with a gram‐negative cell wall Coxiella burnetti.^1^ Q fever may present as an acute illness (such as an influenza‐like illness) or as a chronic disease. Chronic Q fever includes manifestations of endocarditis, vascular infections, osteoarticular infections, chronic hepatitis, glomerulonephritis, and chronic pulmonary infections. Given its multisystem involvement and variable symptom manifestations, Q fever is a diagnosis that can be missed or diagnosed late. Diagnosis of Q fever is largely based on antibodies in serology testing or demonstration of Coxiella burnetti on tissue specimen.

## 2 CASE HISTORY/EXAMINATION

A 77‐year‐old male had a series of presentations with fever of unclear source. His background medical history included stage II chronic kidney disease, ischemic heart disease managed with coronary artery bypass grafts, porcine aortic valve replacement, chronic obstructive lung disease (COPD), paroxysmal supraventricular tachycardia, nonalcoholic steatohepatitis, cholecystectomy, gout, and helicobacter pylori infection. He was a carrier for hemochromatosis. His regular medications were aspirin, citalopram, metoprolol, pantoprazole, and inhalers containing fluticasone propionate, salmeterol and tiotropium. He was an ex‐smoker with a 90‐pack‐year history of smoking and was independent in his activities of daily living. He was living in a rural area with farms in the near vicinity.

On his first presentation, his main symptoms were fevers, productive cough and shortness of breath. His physical exam was largely unremarkable. He had serum creatinine of 94 μmol/L (ref 64–104) and mild hyponatremia with sodium 130 mmol/L (ref 136–145). On his liver function tests he had normal bilirubin, raised gamma‐glutamyl transferase 468 unit/L (ref 12–64) and alkaline phosphatase 391 unit/L (ref 40–150) and a mild transaminitis with alanine transaminase 48 unit/L (ref 0–55) and aspartate aminotransferase 96 unit/L (ref 5–34). He also had raised C‐reactive protein 14.0 mg/L (ref <5.0), mild anemia with hemoglobin 107 g/L (ref 130–170) and neutropenia with neutrophils 1.7 × 10^9^/L (ref 2.0–7.0). His sputum and blood cultures were negative for growth of organisms. His chest X‐ray did not demonstrate any consolidation and he was treated for an infective exacerbation of COPD with antibiotics. Ultrasound abdomen demonstrated hepatomegaly (18.8 cm craniocaudally) with diffuse increase in echogenicity suggesting mild fatty infiltration, absence of intra or extrahepatic ductal dilatation and splenomegaly (19.1 cm craniocaudally). The patient was referred to gastroenterology for follow up of the hepatosplenomegaly.

He presented 6 months later with fevers, weight loss and reduced appetite. His physical exam was unremarkable except for hepatosplenomegaly. He had a raised creatinine 120 μmol/L (ref 64–104), worsening hyponatremia with sodium 124 mmol/L (ref 136–145), raised C‐reactive protein 15.6 mg/mL (ref <5.0) and worsening anemia with hemoglobin 93 g/L (ref 130–170). His hemolysis markers were positive with elevated reticulocyte count 195 × 10^9^/L (ref 50‐100), reduced haptoglobin 0.17 g/L (ref 0.14–2.38) and positive direct antiglobulin test (IgG). On autoimmune screen he had normal complement C3 and C4 levels, positive rheumatoid factor 663 units/mL (ref 0–30), positive anti‐citrullinated peptide autoantibodies (52, ref <5), strongly positive perinuclear antineutrophil cytoplasmic antibodies (p‐ANCA) (1: 640) with Proteinase 3 (PR3) antibody titre 3 (ref <2) and myeloperoxidase (MPO) antibody titre <1 (ref <6). His blood cultures, sputum culture, stool culture and urine culture were negative for growth of organisms.

There was concern for malignancy given the clinical history, cytopenia and splenomegaly and therefore a fluorodeoxyglucose (FDG)‐positron emission tomography (PET) scan (from vertex to mid thighs) was performed that demonstrated nonspecific diffuse hepatosplenomegaly, mediastinal and hilar adenopathy and did not reveal any features of lymphoma nor metastatic disease. A transoesophgeal echocardiogram did not reveal any valvular vegetations. For further investigation of the cytopenia, a bone marrow biopsy was carried out and demonstrated hypercellularity with no cytogenetic abnormalities. He was subsequently discharged from hospital with outpatient follow up organized.

**FIGURE 1:**
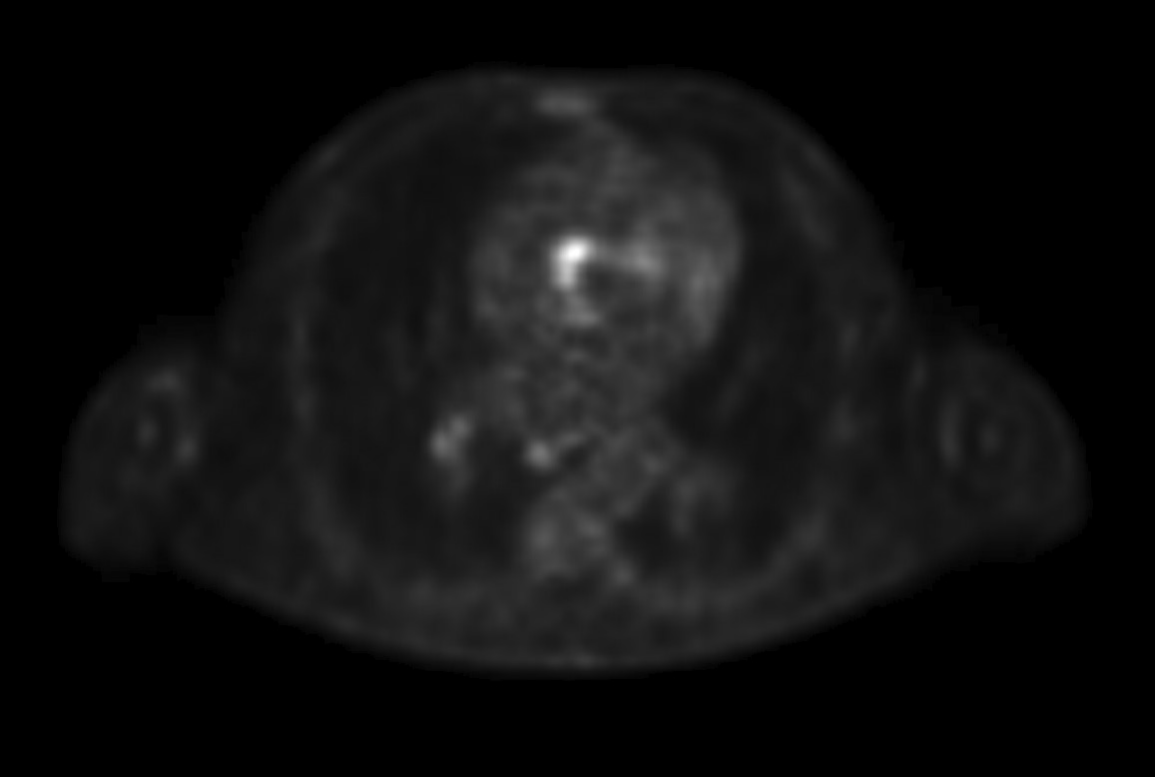
Fluorodeoxyglucose‐positron emission tomography scan demonstrating low grade uptake in the heart in vicinity of aortic‐mitral valve rings. At this time, the transesophageal echocardiogram did not demonstrate any valvular vegetations.

He subsequently re‐presented 3 months later with fevers, confusion and lethargy. He was commenced on empirical antibiotics. He had an acute kidney injury on admission with creatinine 156 μmol/L (ref 64–104) without any hematuria and a slightly raised albumin:creatinine ratio of 8.0 mg/mmol (ref 0.0–3.0). Investigations including urine culture, blood cultures, HIV, Hepatitis B and Hepatitis C serology, cytomegalovirus serology and arbovirus serology (rickettsia, Ross River virus, Barmach Forest virus and Brucella) were negative. He underwent computed tomography (CT) scan of the chest, abdomen and pelvis which revealed hepatosplenomegaly and chest lymphadenopathy but no source of infection. Repeat FDG‐PET scan showed an equivocal low‐grade uptake in the heart in vicinity of aortic‐mitral valve rings (Figure 1). A transoesophgeal echocardiogram did not reveal any valvular vegetations. Flow cytometry did not reveal any clonal populations. Three weeks into the admission the Q fever serology results came back positive (Table 1) and he was commenced on oral doxycycline with clinical improvement and defervescence. Renal function returned to baseline at discharge with serum creatinine 108 μmol/L (ref 64–104).

**TABLE 1:** Q fever serology initially and after 3 months. Reported by the Australian Rickettsial Reference Laboratory, Douglas Hocking Research Institute, The Geelong Hospital.

Q fever serology	Initial titres	3 months later	Reference
QF IgM Index	2.8		<1.0
QF IgA ph 2 IF	> =3200	<25	<25
QF IgM ph 2 IF	> =3200	> =3200	<25
OF IgG ph 2 IF	> = 200	> =3200	<25
QF Total ph 2 IF	> =3200	> =3200	<25
QF IgA ph 1 IF	<25	<25	<25
QF IgM ph 1 IF	800	> =3200	<25
QF IgG ph 1 IF	> =3200	> =3200	<25
QF Total ph 1 IF	> =3200	> =3200	<25

Abbreviations: IF, Immunofluorescence; Ph, Phase; QF, Q fever.

Two months later, the patient was re‐admitted with an acute kidney injury with serum creatinine of 262 μmol/L (ref 64–104). The urinalysis showed microscopic hematuria and dysmorphic red blood cells consistent with glomerular hematuria. He had sub‐nephrotic proteinuria 520 mg/day (ref 0–140). Other investigations included a positive cANCA (1:1280) without detection of MPO and PR3 antibodies, positive double‐stranded deoxyribonucleic acid (dsDNA) antibodies 10 U/mL (ref <7), no paraprotein on immunoelectrophoresis, negative anti‐glomerular basement membrane antibodies, negative cryoglobulin, negative extractable nuclear antigens screen and normal C3 and C4 levels.

## 3 METHODS (DIFFERENTIAL DIAGNOSIS, INVESTIGATION, AND TREATMENT)

**FIGURE 2:**
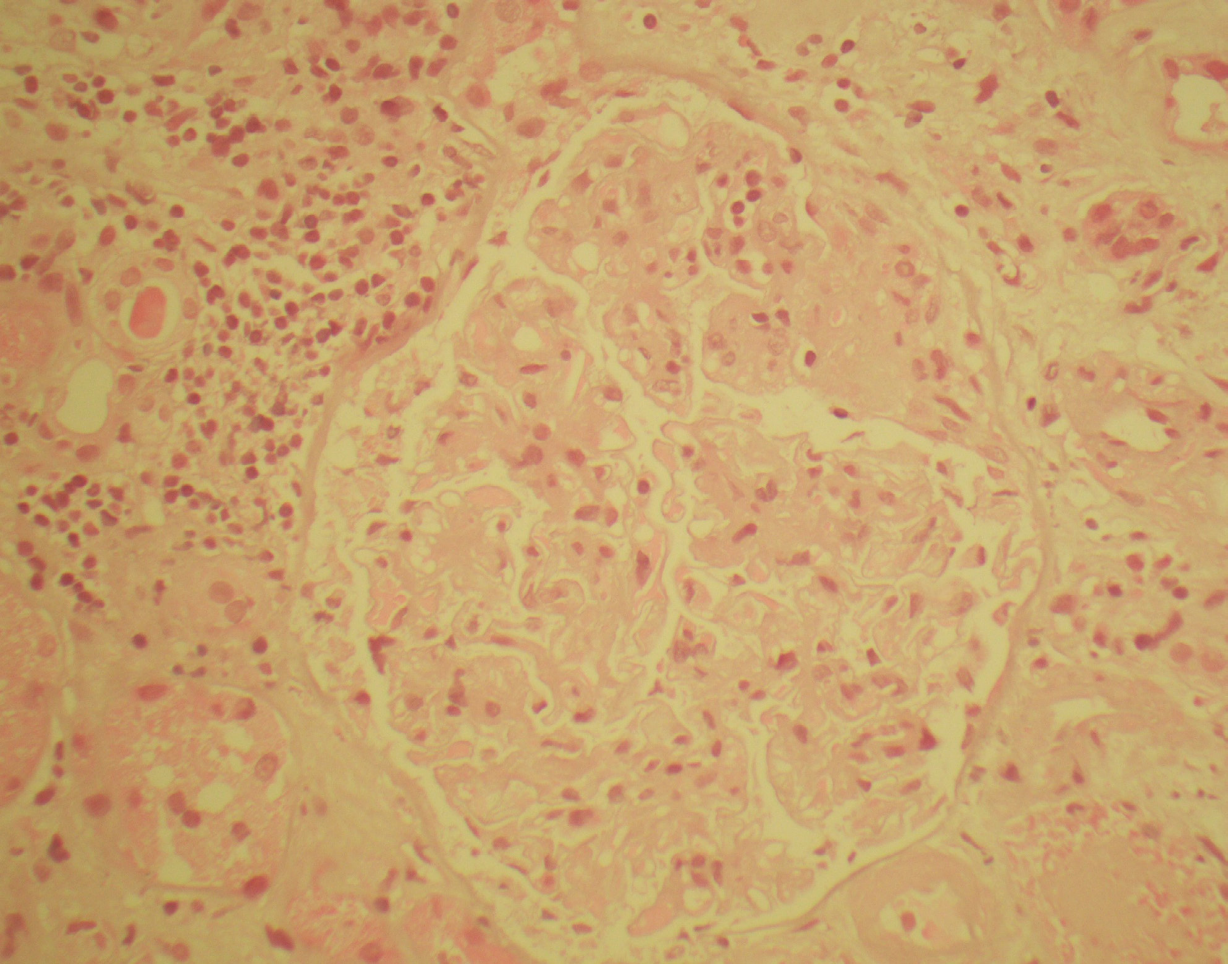
Light microscopy from renal biopsy specimen. Hematoxylin and eosin stain at 400× magnification. A glomerulus with a mild increase in mesangial matrix, a small focus of endocapillary hypercellularity and a small focus of segmental sclerosis.

The renal biopsy demonstrated a mesangiopathic pattern on light microscopy (Figure 2; Appendix S1). The glomeruli had a mild to moderate increase in mesangial matrix without mesangial hypercellularity. Several glomeruli also had segmental endocapillary proliferation with obscuration of capillary lumens by mononuclear cells. One of these endocapillary lesions had an associated capsular adhesion and small focus of segmental sclerosis. Otherwise, occasional glomeruli also had some ischemic capillary wall wrinkling and tuft shrinkage. There was no evidence of fibrinoid necrosis or crescents. There was moderate background chronic tubulointerstitial atrophy/fibrosis and mild chronic interstitial inflammatory cell infiltrate. Several red blood cell casts were seen. The arterioles had mild hyaline change without any evidence of vasculitis. On immunofluorescence the glomeruli had positive staining (more granular mesangial than the capillary wall) for IgM (2–3+, on a 0–3 scale), C1q (2+), C3 (1+), kappa (1+), lambda (2+), and negative staining for IgG, IgA and fibrinogen. On electron microscopy there was mild focal thickening of glomerular basement membranes, a mild focal increase in mesangial matrix, mild focal effacement of foot processes and no dense deposits or abnormal fibrils were seen in the glomerulus. Polymerase chain reaction (PCR) of Coxiella genes (*Com1* & *htpAB*) in the biopsy specimen was negative. Lupus nephritis was considered unlikely given normal complement levels in serum and lack of IgG staining on immunofluorescence. The main differential diagnoses considered were an infection‐associated glomerulonephritis and C1q nephropathy.

## 4 CONCLUSION AND RESULTS (OUTCOME AND FOLLOW‐UP)

A follow‐up transoesophageal echocardiogram demonstrated an independently mobile tissue‐density echogenic vegetation measuring 9 mm × 3 mm on the bioprosthetic aortic valve and severe paravalvular leak. Based on the modified Duke Criteria, a diagnosis was reached for infective endocarditis secondary to Coxiella burnetti.^2^ Due to comorbidities, the patient was not deemed suitable for surgical intervention, subsequently deteriorated and was transitioned to a supportive care pathway under palliative care.

## 5 DISCUSSION

There are several published case reports of renal disease in patients with chronic Q fever including diffuse proliferative glomerulonephritis, focal and segmental proliferative glomerulonephritis, and membranoproliferative glomerulonephritis.^3^, ^4^, ^5^, ^6^ Urinary abnormalities such as hematuria, proteinuria and sterile pyuria are also not uncommon in this patient population.^7^, ^8^, ^9^

Our patient likely developed a proliferative glomerulonephritis during the period of his acquisition of acute Q fever and its transformation to chronic Q fever indicating a strong temporal correlation. The presence of other autoantibodies (dual ANCA positivity, rheumatoid factor, anti‐citrullinated peptide autoantibodies, anti‐dsDNA antibodies) and positive Coomb's test positive in our patient indicates that possibly the abnormal autoimmune response led to the development of an immune‐mediated glomerulonephritis. Aberrant immune response is well known phenomenon in Q fever and various autoantibodies including dual ANCA positivity, anti‐nuclear antibodies, anti‐dsDNA antibodies, anti‐smooth muscle antibodies, anti‐cardiac muscle antibodies, and anti‐phospholipid antibodies have been demonstrated in patients with Coxiella burnetti infection.^10^, ^11^, ^12^ The significance of this immunological response remains unknown.

The secondary glomerulonephritis entity in our case presents several diagnostic possibilities.

C1q nephropathy was first described by Jennette and Hipp in 1985.^13^ It is defined by the presence of extensive mesangial deposits of C1q with associated mesangial immunoglobulins and the absence of evidence of systemic lupus erythematosus. The light microscopy findings are heterogeneous and can vary from proliferative to nonproliferative glomerulonephritis. C1q nephropathy usually presents with heavy proteinuria but rapid deterioration of renal function and crescentic transformation has been described in literature.^14^, ^15^, ^16^ Secondary forms of C1q nephropathy have been described due to various viral infections.^17^ There are still uncertainties on further investigations and management of this clinical entity.^18^ Our case demonstrates codominant C1q staining which would be consistent with a diagnosis of C1q nephropathy. However, the lack of dense deposits on electron microscopy distracts from this diagnosis. No cases of Cq1 nephropathy associated with Q fever have been reported to our knowledge.

An alternative diagnostic possibility is infection‐related glomerulonephritis. This would be consistent with the endocapillary proliferation seen in several of the glomeruli and temporal association with infection. However, against this diagnosis are the normal complement levels and the unusual C1q deposition.

The lack of PCR detection of Coxiella burnetti in the biopsy specimen is of unclear significance. PCR can be used as a detection method in clinical samples for diagnosis of Q fever however there can be false negatives.^1^ Previous case reports of Q fever‐associated glomerulonephritis have not tested for PCR of Coxiella genes. The lack of detection of Coxiella genes in our case suggests the development of glomerulonephritis is driven by autoimmune dysfunction rather than direct invasion of renal tissue.

In summary, secondary glomerulonephritis can develop as a complication of chronic Q fever infection. This report adds another facet to the possible immunological complications associated with the Coxiella burnetti infection.

## AUTHOR CONTRIBUTIONS

**Craig Peter Coorey:** Writing – original draft; writing – review and editing. **William James:** Supervision; writing – review and editing. **Venkat Manickavasagam:** Supervision; writing – review and editing. **Jason Chen:** Resources; writing – review and editing. **Sanjeev Baweja:** Conceptualization; supervision; writing – original draft; writing – review and editing.

## CONFLICT OF INTEREST STATEMENT

The authors declare no conflicts of interest.

## FUNDING STATEMENT

Open access publishing facilitated by Western Sydney University.

## CONSENT

Written informed consent for the publication of this report from the patient's next of kin.

## Supporting information


Appendix S1.

## Data Availability

The data that supports the findings of this study are available within the article and in the supplementary material of this article.
