# Supplementary material for: Endocapillary proliferative glomerulonephritis associated with Q fever endocarditis: A case report
Source: Clin Case Rep. 2024 Oct 21;12(10):e9473. doi: 10.1002/ccr3.9473 (PMC11491761; doi:10.1002/ccr3.9473)
Supplement: Supplementary file 1 — Appendix S1. [file CCR3-12-e9473-s001.docx]

**Appendix**

Renal Biopsy Pathology Report

MACROSCOPIC:

The specimen consists of 2 tan tissue cores 13 and 11 mm. The cores are sampled for IF and EM, and the remainder of the specimen is all embedded for paraffin sections in 1 block.

MICROSCOPIC:

The renal biopsy consists predominantly (60%) of cortex, together with some (40%) medulla. 17 glomeruli are present, 3 of which are obsolescent in keeping with age.

The glomeruli have a mild to moderate increase in mesangial matrix, without mesangial hypercellularity. A couple of glomeruli also have segmental endocapillary proliferation, with obscuration of capillary lumens by mononuclear cells. One of these endocapillary lesions has an associated capsular adhesion and/or small focus of segmental sclerosis. Otherwise, occasional glomeruli also have some ischaemic capillary wall wrinkling and tuft shrinkage. The capillary walls have the occasional suggestion of some thickening. No definite double contours are seen on the silver stain. There is no evidence of fibrinoid necrosis or crescents.

There is at least moderate background chronic tubulointerstitial atrophy/fibrosis. There is a mild chronic interstitial inflammatory cell infiltrate. The tubules have moderate degenerative/regenerative changes and occasional vacuolisation. The proximal tubules have prominent intracytoplasmic hyaline droplets, suggestive of proteinuria. Several red blood cell casts are seen.

The arterioles have mild hyaline change. There arteries have moderate arteriosclerosis, with intimal fibrosis and reduplication of the internal elasti lamina. The arterioles and arteries have no evidence of vasculitis. The amyloid stain (Congo red) is negative.

IMMUNOFLUORESCENCE:

A further 2 viable glomeruli are seen in the immunofluorescence sections.

IgA: negative to trace staining.

IgG: negative to trace staining.

IgM: moderate to strong (2-3+) granular mesangial >> capillary wall staining.

C1q: moderate (2+) granular mesangial >> capillary wall staining.

C3: weak (1+) granular mesangial >> capillary wall staining.

Kappa: weak (1+) granular mesangial >> capillary wall staining.

Lambda: moderate (2+) granular mesangial >> capillary wall staining.

Fibrinogen: negative.

COMMENT/SUMMARY:

The kidney has a mesangiopathic pattern, together with moderate/strong mesangial staining for C1q and IgM. 2 of 14 viable glomeruli have segmental endocapillary proliferation, 1 also with an association small focus of segmental sclerosis. No crescents are seen. The features are best placed in the entity of 'C1q nephropathy'. An infection-associated glomerulonephritis remains possible, however the C1q staining seen is unusual. Lupus neutrophils is not favoured due

to the lack of IgG staining.

There is significant background chronic disease, with at least moderate background chronic tubulointerstitial atrophy/fibrosis and moderate arteriosclerosis.

ELECTRON MICROSCOPY:

One glomerulus was examined by electron microscopy.

The glomerulus shows mild nonspecific changes. There is mild focal thickening of glomerular basement membranes, a mild focal increase in mesangial matrix and mild focal effacement of foot processes. Podocytes have microvilli. Endothelium appears normal. No dense deposits or abnormal fibrils are seen. There is mild interstitial fibrosis accompanied by a focal lymphocytic infiltrate. Some tubule cells have lipofuscin granules.

ELECTRON MICROSCOPY COMMENT:

The changes seen in this specimen are mild and nonspecific. No dense deposits or abnormal fibrils are seen in the glomerulus.

ELECTRON MICROSCOPY SUMMARY:

RENAL BIOPSY (EM): Mild nonspecific changes.

ADDITIONAL REPORT:

Coxiella burnetii (Q-fever) PCR

RT-PCR result: NOT DETECTED
